# ^68^Ga-Sienna+ for PET-MRI Guided Sentinel Lymph Node Biopsy: Synthesis and Preclinical Evaluation in a Metastatic Breast Cancer Model

**DOI:** 10.7150/ntno.34727

**Published:** 2019-06-06

**Authors:** Heli Savolainen, Alessia Volpe, Alkystis Phinikaridou, Michael Douek, Gilbert Fruhwirth, Rafael T. M. de Rosales

**Affiliations:** 1 Department of Imaging Chemistry and Biology, School of Biomedical Engineering & Imaging Sciences, King's College London, London, United Kingdom; 2 Department of Biomedical Engineering, School of Biomedical Engineering & Imaging Sciences, King's College London, London, United Kingdom; 3 Department of Research Oncology, School of Cancer & Pharmaceutical Sciences, King's College London, London, United Kingdom; 4 London Centre for Nanotechnology, King's College London, Strand Campus, London, WC2R 2LS, United Kingdom (UK).

**Keywords:** ^68^Ga, PET-MRI, sentinel lymph node biopsy, SPIO

## Abstract

Sentinel lymph node biopsy (SLNB) is commonly performed in cancers that metastasise *via* the lymphatic system. It involves excision and histology of sentinel lymph nodes (SLNs) and presents two main challenges: *(i)* sensitive whole-body localisation of SLNs, and *(ii)* lack of pre-operative knowledge of their metastatic status, resulting in a high number (>70%) of healthy SLN excisions. To improve SLNB, whole-body imaging could improve detection and potentially prevent unnecessary surgery by identifying healthy and metastatic SLNs. In this context, radiolabelled SPIOs and PET-MRI could find applications to locate SLNs with high sensitivity at the whole-body level (using PET) and guide high-resolution MRI to evaluate their metastatic status. Here we evaluate this approach by synthesising a GMP-compatible ^68^Ga-SPIO (^68^Ga-Sienna+) followed by PET-MR imaging and histology studies in a metastatic breast cancer mouse model.

**Methods.** A clinically approved SPIO for SLN localisation (Sienna+) was radiolabelled with ^68^Ga without a chelator. Radiochemical stability was tested in human serum. *In vitro* cell uptake was compared between 3E.Δ.NT breast cancer cells, expressing the hNIS reporter gene, and macrophage cell lines (J774A.1; RAW264.7.GFP). NSG-mice were inoculated with 3E.Δ.NT cells. Left axillary SLN metastasis was monitored by hNIS/SPECT-CT and compared to the healthy right axillary SLN. ^68^Ga-Sienna+ was injected into front paws and followed by PET-MRI. Imaging results were confirmed by histology.

**Results.** ^68^Ga-Sienna+ was produced in high radiochemical purity (>93%) without the need for purification and was stable *in vitro*. *In vitro* uptake of ^68^Ga-Sienna+ in macrophage cells (J774A.1) was significantly higher (12 ± 1%) than in cancer cells (2.0 ± 0.1%; *P* < 0.001). SPECT-CT confirmed metastasis in the left axillary SLNs of tumour mice. In PET, significantly higher ^68^Ga-Sienna+ uptake was measured in healthy axillary SLNs (2.2 ± 0.9 %ID/mL), than in metastatic SLNs (1.1 ± 0.2 %ID/mL; *P* = 0.006). In MRI, ^68^Ga-Sienna+ uptake in healthy SLNs was observed by decreased MR signal in T2/T2*-weighted sequences, whereas fully metastatic SLNs appeared unchanged.

**Conclusion.** ^68^Ga-Sienna+ in combination with PET-MRI can locate and distinguish healthy from metastatic SLNs and could be a useful preoperative imaging tool to guide SLN biopsy and prevent unnecessary excisions.

## Introduction

Several cancers such as melanoma, breast and prostate cancer are known to spread through the lymphatic system 1. Sentinel lymph nodes (SLN) are described as the first nodes to receive lymphatic drainage from a tumour and sentinel lymph node biopsy (SLNB) involves the identification of SLNs for removal and histopathological analysis to evaluate the presence or absence of metastases. Based on this information, clinicians decide whether or not further surgery is indicated. Surgical removal of metastatic SLNs and SLNB can improve disease-free interval and increase survival rate 2,3. However, >70% of excised SLNs in breast cancer or melanoma have been found to be negative/healthy SLNs, exposing these patients to unnecessary surgery and post-operative risks such as lymphoedema, seroma formation and nerve or vessel damage 4. Thus, a non-invasive preoperative imaging method of locating and discriminating between metastatic and healthy SLNs would be an important tool to avoid unnecessary biopsies and improve guidance for SLNB_._

Several clinical imaging methods are available for the detection of SLNs. Ultrasound and computed tomography (CT) have been used to find enlarged SLNs, but size is not a suitable indicator for metastasis 5. ^99m^Tc-nanocolloid mixed with a blue/fluorescent dye can be used for SLN imaging by lymphoscintigraphy or single-photon emission computed tomography (SPECT) 6. However, these imaging techniques only locate SLNs, and do not detect the presence of metastasis. [^18^F]FDG positron emission tomography (PET) is useful to identify macrometastases but not micrometastases (size between 0.2 - 2 mm) and it may be difficult to locate metastases in LNs in close proximity to areas of high [^18^F]FDG uptake 7. Dynamic [^18^F]FDG PET-lymphography is a recent promising technique where the radiotracer is injected subcutaneously, and has been shown to be efficient at locating and characterizing SLNs but lacks sensitivity in differentiating acute inflammatory lymphadenopathy from metastatic LNs 8. Photoacoustic imaging has also shown promise for detecting LN micrometastases in melanoma 9,10.

MRI has excellent spatial resolution and, in combination with superparamagnetic iron oxide nanoparticles (SPIOs), is capable of detecting micrometastases in the clinical setting at very low SPIO concentrations 11,12. SPIOs are taken up by macrophages where they accumulate creating negative contrast (low signal intensity in T2/T2* weighted MRI) in the healthy tissue of SLNs, but not in metastatic LN tissue that lacks high concentrations of macrophages 4. This property has resulted in the identification of contrast patterns of SLN uptake for metastatic and non-metastatic nodes 13. The low sensitivity of MRI, however, makes whole-body identification of SPIO biodistribution and SLN localisation challenging, which is an important consideration in cancers with distant SLNs and/or variable location of the primary tumour, such as melanoma 14.

**Scheme 1:**
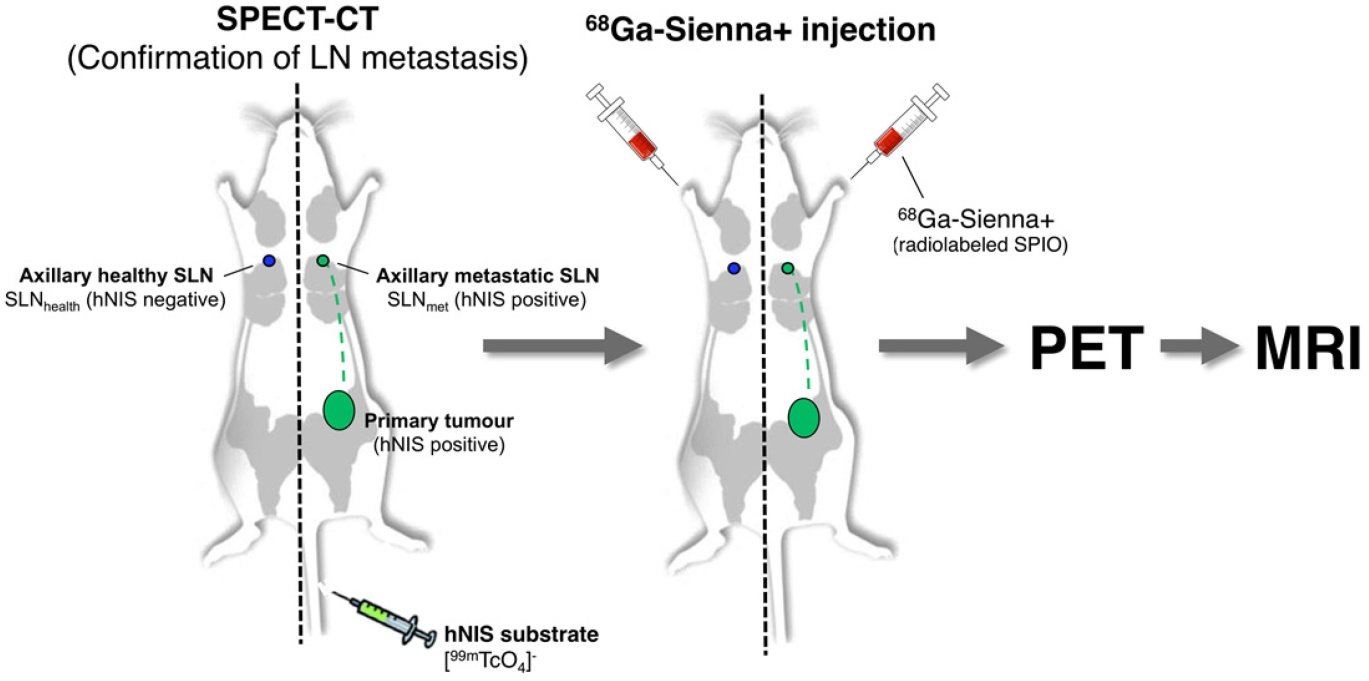
Schematic representation of the experimental protocol used to evaluate the potential of ^68^Ga-Sienna+ for PET-guided MR imaging of sentinel lymph node biopsy.

One approach to overcome this limitation of SPIO-MRI is to combine the full-body imaging capabilities and sensitivity of PET with the ability of SPIO-MRI to identify SLN metastases using radiolabelled SPIOs. This approach would allow the use of PET for both sensitive whole-body SLN localisation and also guiding high resolution MRI for evaluating intra-LN SPIO distribution. Several groups, including ours, have previously demonstrated the concept of SLN localisation with radiolabelled SPIOs using nuclear-MR imaging; however, to the best of our knowledge, none have demonstrated localisation and characterisation in a metastatic animal cancer model 15-28. The aim of this work was to develop a clinically translatable ^68^Ga-labelled PET-MR SLN imaging agent based on a CE-approved SPIO for SLN localisation (Sienna+). To evaluate its potential in a relevant metastatic mouse cancer model, we chose a reporter gene (human sodium iodide symporter - hNIS) expressing breast cancer mouse model that develops spontaneous axillary lymph node metastases and allows the use of SPECT imaging with ^99m^TcO_4_^-^ (hNIS substrate) to confirm the health status of SLNs prior to ^68^Ga-Sienna+ PET-MRI (Scheme 1).

## Materials and Methods

### Radiolabelling of Sienna+ with ^68^Ga

Sienna+ SPIO nanoparticles with carboxydextran coating (26-30 mg Fe/mL, Z-average diameter 59 ± 0.7 nm; pH = 5.0-7.0; sterile) were purchased from Endomag (Cambridge, UK). ^68^Ga *(t_1/2_* = 68 min, β^+^ = 89%) was eluted from a ^68^Ge/^68^Ga-generator (Eckert & Ziegler, Germany) with 5 mL of 0.1 M HCl for high performance capillary electrophoresis (HPCE) (Fluka, USA). The pH of the gallium elution (400 µL) was adjusted to 5 with 4 M ammonium acetate solution (40 µL, 160 µmol, Sigma-Aldrich, UK), Sienna+ (67 µL, 2 mg Fe) was then added and the mixture reacted in a sealed 1.5 mL plastic microcentrifuge tube at 100 °C for 10 min. The reaction solution was filtered (sterile hydrophilic Millex-LG 13 mm 0.2 µm PTFE, Merck, Germany). The pH of the final product was ~6. Radiolabelling using GMP protocol: A CE-approved sterile vial of Sienna+ (2 mL, 60 mg Fe) was injected with 100 µL of the pH-adjusted ^68^Ga elution (pH 5) using sterile techniques with a 1 mL syringe and filter (same as above). The vial was incubated at 100 °C for 10 min. After cooling, the radiochemical purity of ^68^Ga-Sienna+ was measured using TLC (F-254 silica gel 60 plates, Merck) with citrate buffer (pH 5) as an eluent (0.1 M tri-sodium citrate dihydrate (Sigma-Aldrich) and 0.1 M citric acid monohydrate (Fisher Scientific, UK)) and analysed by autoradiography. Exposed phosphor screens (PerkinElmer, USA) were scanned with a Cyclone Plus system (PerkinElmer). The percentage of ^68^Ga-Sienna+ and free ^68^Ga were calculated by region of interest (ROI) analysis using OptiQuant 5.0 software (PerkinElmer). Nanoparticle hydrodynamic size was measured by dynamic light scattering (DLS) and the effective electric charge of the surface by zeta potential measurements in a Zetasizer Nano ZS instrument (Malvern Instruments Ltd, UK) in deionised water (size) or 10% PBS (zeta potential).

### *In vitro* serum stability

^68^Ga-Sienna+ (100 µL, 10 MBq, 0.3 mg Fe) was mixed with human serum (900 µL, Sigma-Aldrich) and the mixture incubated at 37 °C for 4 h. Samples (200 µL) were collected every hour and analysed by size-exclusion chromatography with a Superose 6 Increase 10/300 GL column (GE Healthcare, UK) connected to an Äkta system (GE Healthcare) using a 0.5 mL/min flow of PBS as eluent. Samples of 1 mL were collected and measured in a gamma counter (LKB Wallac, Finland). ^68^Ga-Sienna+ eluted at 8-15 mL and serum-bound/free ^68^Ga at 20-25 mL. Serum stability was expressed as percentage of ^68^Ga-Sienna+ in serum (as calculated from size-exclusion chromatography) over time.

### Cell culture and *in vitro* uptake studies

Rat breast adenocarcinoma cells MTLn3E Δ34-CXCR4-GFP hNISTagRFP (3E.Δ.NT) were generated and characterised as previously described 29. Mouse monocyte macrophages J774A.1 were a gift from Dr. Varsha Kanabar at the Institute of Pharmaceutical Sciences in King's College London. Generation and characterisation of mouse leukaemic monocyte macrophage RAW264.7.GFP cells are described in the supplemental information. All cell lines were maintained in an atmosphere of 5% CO_2_/95% air. All cell culture reagents were obtained from Sigma-Aldrich. 3E.Δ.NT cells were cultured in MEM eagle with alpha modification supplemented with 5% FBS, 100 IU penicillin-streptomycin, 0.5 mg/mL G 418 disulfate salt and 0.5 µg/mL puromycin. Macrophage cell lines were cultured in DMEM high glucose medium supplemented with 10% FBS and 1 mM sodium pyruvate. All cell media were supplemented with 2 mM L-glutamine.

For cell uptake studies of ^68^Ga-Sienna+, each cell line was cultured on 6-well plates 24 h before the experiment using 1 million cells/well. The medium was then removed and 1 mL of ^68^Ga-Sienna+ solution in complete medium was added (600 kBq/mL, 5.3 µg iron/mL). Cells were incubated for 1, 2 or 3 h. The supernatant was collected and cells washed twice with PBS. 3E.Δ.NT cells were collected by trypsination. Macrophages were collected by scraping. The radioactivity of the samples (cell pellet, supernatant and washings) was measured in a gamma counter to allow us to calculate cell uptake, expressed as a percentage of total radioactivity per million cells.

### Animal experiments

Animal experiments were approved by the King's College London Animal Welfare and Ethical Review Body (AWERB) and were in agreement with UK Home Office regulations. Female 5-6 weeks old NOD scid gamma (NSG) (NOD.Cg-*Prkdc^scid^ Il2rg^tm1Wjl^/*SsJ, n = 6) and B6CBAF1 mice were obtained from Charles River (UK). Mice were housed within filter-top cages at least 5 days before starting the experiments and given food and water *ad libitum*. For tumour inoculation, 3E.Δ.NT cells (1 × 10^6^ cells in 50 µL of PBS) were injected subcutaneously into the left mammary fat pad between the fourth and fifth nipple in the NSG mice. Once tumours were palpable, their volume was monitored using calipers. During tumour inoculation and imaging procedures, mice were anaesthetised with isoflurane (Vet Tech Solutions Ltd., UK), 3% in oxygen for induction and 1.5-2% for maintenance.

### SPECT-CT and PET-CT imaging

On day 19 or 20 after inoculation, NSG mice were scanned with SPECT-CT using ^99m^Tc-pertechnetate (^99m^TcO_4_^-^) to confirm metastasis of left axillary SLNs. ^99m^TcO_4_^-^ was eluted from a Drytec generator (GE Healthcare) using saline. ^99m^TcO_4_^-^ (100 µL, 31 ± 3 MBq) was injected intravenously into the tail vein. After 40 min, a 30 min scan was acquired on a NanoSPECT-CT Silver Upgrade (Mediso, Hungary) with 1 mm collimators, 45 s/frame. CT was obtained with 55 kVp tube voltage, 1000 ms exposure time and 180 projections.

A healthy B6CBAF1 mouse was used to find the optimal time point for a PET scan after ^68^Ga-Sienna+ injection (20 µL, 2.2 ± 0.9 MBq, 79 µg Fe) subcutaneously into left front paw. A scan was acquired for 2.5 h on a nanoScan PET-CT preclinical scanner (Mediso) 20 min after injection. CT was obtained with a 45 KVp tube voltage, 600 ms exposure time and 180 projections.

For the tumour/SLN metastasis study NSG mice were imaged by PET-CT on day 21/22 after inoculations. ^68^Ga-Sienna+ (20 µL, 2.2 ± 0.9 MBq, 79 µg Fe) was injected subcutaneously into both left and right front paws. After 1 h, a 30 min scan was acquired. SPECT-CT images were reconstructed in a 128 × 128 matrix using HiSPECT (Sci-vis GmbH, Germany) software package, and images fused using Bioscan InVivoScope (Bioscan, USA) software. PET-CT data were reconstructed using a Monte Carlo based full 3D iterative algorithm (Tera-Tomo, Mediso). Data were corrected for attenuation and scatter, and decay correction for the time of injection was applied. A total of 4 iterations and 6 subsets were applied. SPECT-CT Images were reconstructed with a voxel size of 0.16 x 0.16 x 0.16 mm and PET scans with a voxel size of 0.21 x 0.21 × 0.21 mm. Images were analysed using VivoQuant 2.50 software (Invicro LLC., USA) by drawing ROIs and calculating %ID/mL values.

### MR imaging

MR imaging was performed 6 h after ^68^Ga-Sienna+ injection using a 3T Philips Achieva MR scanner (Philips Healthcare, The Netherlands) equipped with a clinical gradient system (30 mTm^-1^, 200 mT/m/ms) and a single-loop surface coil (diameter = 47 mm). Mice were anesthetised and placed on the coil in a prone position. After scout scans, a coronal 3D turbo spin echo T2-weighted scan (TR = 500 ms, TE = 61 ms, flip angle = 90°, FOV = 40 x 30 x 12 mm^3^, acquired matrix 200 x 148, slice thickness = 0.5 mm, resolution = 0.2 x 0.2 mm, slice number = 24, averages = 2, acceleration factor = 10, scan duration = 8 min 49.5 s) and a transverse 3D turbo spin echo T2-weighted scan were acquired (TR = 500 ms, TE = 47 ms, flip angle = 90°, FOV = 31 x 31 x 8 mm, acquired matrix 80 x 74, slice thickness = 0.5 mm, resolution = 0.4 x 0.4 mm, slice number = 16, averages = 4, acceleration factor = 10, scan duration = 6 min 1.5 s). Finally, coronal 3D T2 maps were acquired with a turbo spin echo sequence (TR = 500 ms, TE = 21 ms, echo spacing = 28 ms, 5 echoes, flip angle = 90°, FOV = 25 x 25 x 12 mm, acquired matrix 124 x 124, slice thickness = 0.5 mm, resolution = 0.2 x 0.2 mm, slice number = 24, averages = 1, acceleration factor = 10, scan duration = 18 min 32.5 s) and coronal 3D T2* maps with a turbo gradient echo sequence (TR = 44 ms, TE = 4.7 ms, echo spacing = 5.0 ms, 8 echoes, flip angle = 25°, FOV = 25 x 25 x 12 mm, acquired matrix 124 x 124, slice thickness = 0.5 mm, resolution = 0.2 x 0.2 mm, slice number = 24, averages = 4, acceleration factor = 1, scan duration = 19 min 57.5 s).

MR scans were analysed using OsiriX Lite software (Pixmeo SARL, Switzerland) and ROIs were drawn manually around the axillary LNs on the T2 and T2* images. The ROI was drawn on the first image and then propagated to the rest of the echoes. Signal intensities of 3 consecutive slices containing the LNs were averaged and plotted against echo times. Exponential decay was fitted in GraphPad Prism 7 (USA) using a one-phase decay. Spin-spin relaxation time T2 (ms) was calculated as 1/-K, K being the rate constant, and relaxation rate R2 (s^-1^) as 1/T2x1000. T2* and R2* were calculated the same way.

### Histology

Mice were terminated after MR scans and fluorescence photographs were taken using a Nikon D5200 camera (Japan) and a Nikon DX AF-S NIKKOR 18-55nn 1:3.5-5.6G ED II lens connected to an appropriate emission filter (SFA-LFS-RB, Nightsea, USA) using filter goggles and a fluorescent torch (emission 500 nm). Axillary LNs (metastatic and non-metastatic) were collected for histological analysis. LNs were fixed in 10% neutral buffered formalin (Pioneer Research Chemicals Ltd., UK) for a minimum of 2 days. LNs were processed into paraffin blocks using the Leica Biosystems tissue processor (Germany). Tissues were dehydrated through 70% EtOH 1.5 h, 100 % EtOH 5 h, xylene 3 h and paraffin wax 3 h. Nodes were cut into 5 µm sections and mounted on superfrost plus slides (VWR, USA). Immunohistochemistry staining was performed in the Ventana Discovery XT instrument (Ventana Medical Systems, USA), using the Ventana DAB Map detection Kit. Cancer cells were detected by GFP staining. Sections were pre-treated with EDTA buffer (45 min, Ventana cell condition 1 solution) before incubation in a primary antibody solution (1:5000 ab290 8 h, Abcam, UK) and secondary swine anti-rabbit antibody (1:200 E0353 32 min, Dako, USA). Macrophages were stained with Iba1 antibody (1:250, 4 h, Wako Chemicals GmbH, Germany) in the same way as in GFP staining, except that slides were incubated in the secondary antibody solution for 1 h. All slides were haematoxylin counterstained according to automatic protocol. Iron staining was performed with Perls Prussian blue protocol: sections were brought to distilled water and flooded with equal parts mixture of 2% ferrocyanide (VWR) and 2% hydrochloric acid (VWR) for 10 min. Slides were washed with water and counterstained with 0.1% neutral red stain (Acros Organics, Belgium) for 1 min. Slides were rinsed with water and dehydrated in absolute alcohol. Slides were digitised using the Leica SCN400F scanner with 40x magnification.

### Statistical analysis

Statistical significances (*P* < 0.05) in the cell uptake studies were calculated by one-way ANOVA with Bonferroni correction and otherwise by two-tailed independent samples Student's t-test using IBM SPSS Statistics 24 software (USA).

## Results

### ^68^Ga-radiolabelling of Sienna+

**Figure 1:**
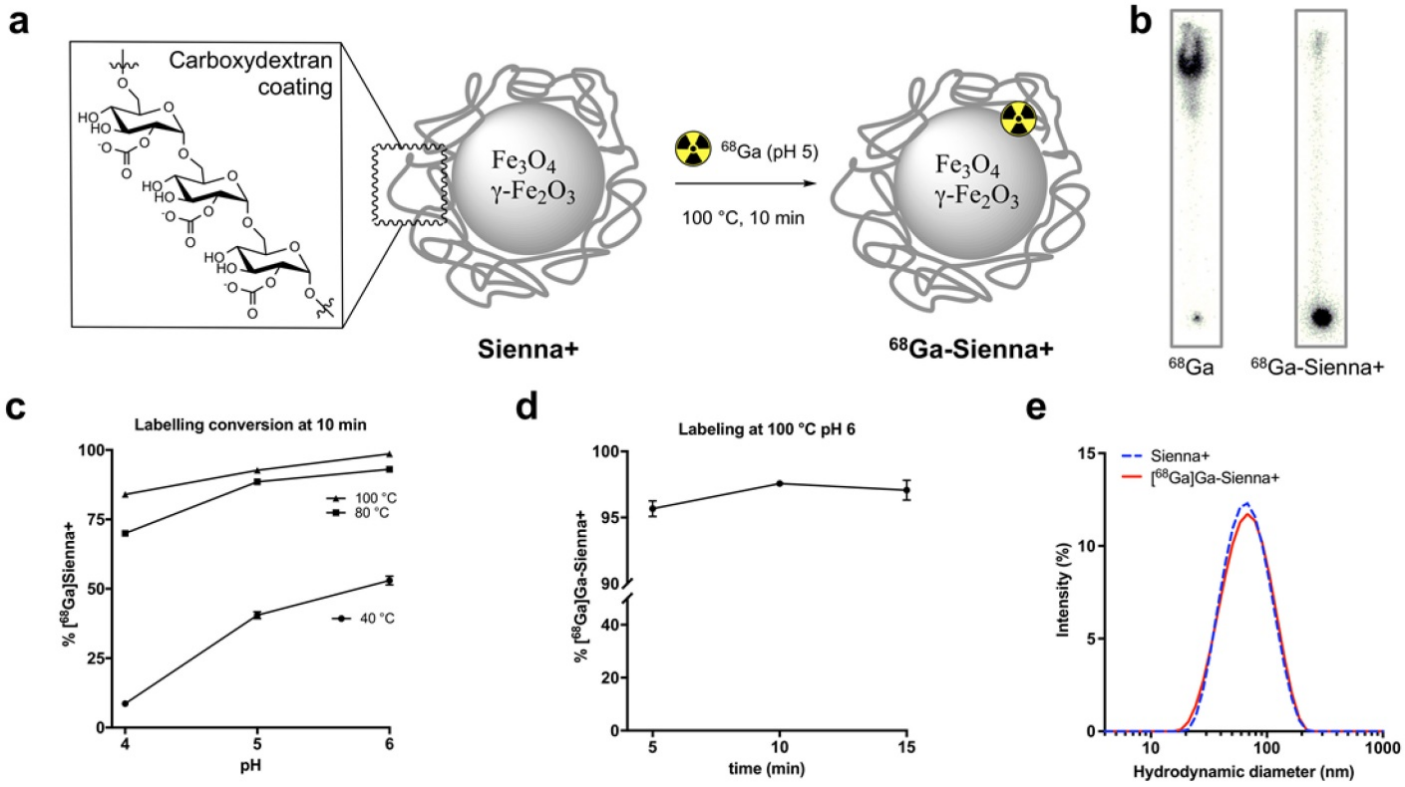
Radiolabelling studies: (**a**) ^68^Ga-radiolabelling of Sienna+, (**b**) Radio-TLC of unchelated ^68^Ga and ^68^Ga-Sienna+; (**c**) Radiolabelling yield optimization at different pH and temperatures (mean ± SEM, n = 3-4); (**d**) Radiolabelling yields at 100 °C pH 6 using different reaction times (mean ± SEM, n = 3); (**e**) DLS measurement comparison of Sienna+ and ^68^Ga-Sienna+ (GMP protocol).

Sienna+ was radiolabelled with ^68^Ga without using a chelator (Figure 1a). The reaction was optimised by testing the effect of pH (4, 5 and 6, before adding Sienna+) and temperature (40, 80 and 100 °C) using a 10 min reaction time (Figure 1c). Analysis of the reaction conversion was performed by radio-TLC. The majority (>95%) of free ^68^Ga eluted with the solvent front, whereas ^68^Ga-Sienna+ stayed at the origin (Figure 1b). Increasing temperature and pH increased radiolabelling conversion (Figure 1c). Highest conversion of ^68^Ga-Sienna+ was obtained at 100 °C at pH 6 in 10 min reaction time (Figure 1d). DLS measurements, however, showed aggregation at these conditions (data not shown, hydrodynamic size >100 nm). Therefore, for the *in vitro/vivo* experiments, Sienna+ was radiolabelled at pH 5 and 100 °C in 10 min yielding ^68^Ga-Sienna+ with 93 ± 0.8 % radiochemical purity (RCP) and in 78 ± 1.4 % radiochemical yield (RCY) (low RCY due to loss of material during the sterile filtration step). The maximum specific activity (A_s_ ) obtained was 67 MBq/mg of Fe (non-optimised and non-decay corrected). Hydrodynamic size was slightly higher and zeta potential lower than for unlabelled Sienna+ (Table 1). However, when the entire contents of the vial were labelled (using GMP-radiolabelling protocols), RCP and RCY >93% were obtained and no changes in hydrodynamic size/zeta potential were observed (Figure 1e, Table 1).

### *In vitro* serum stability and cell uptake studies

**Figure 2:**
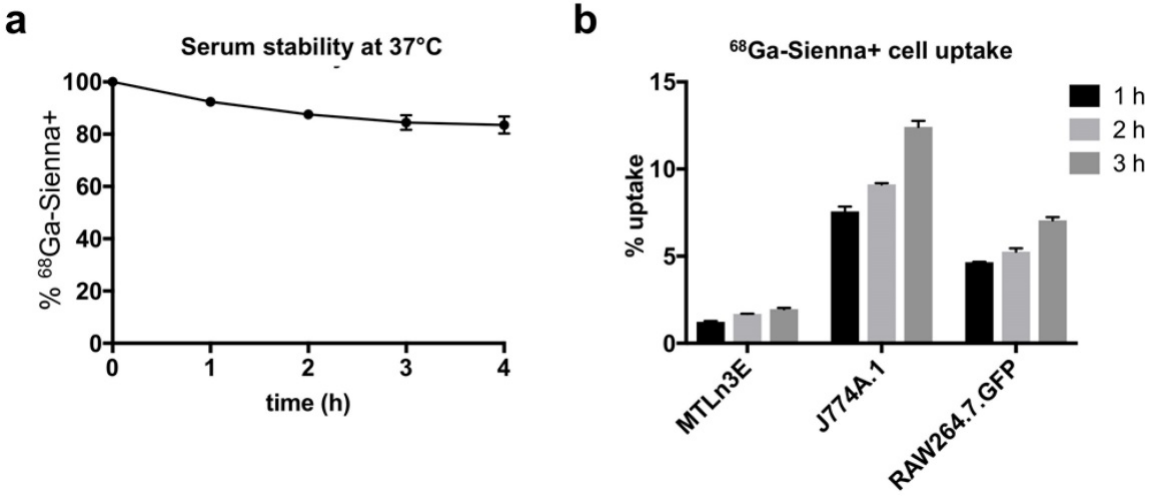
*In vitro* experiments: (**a**) Stability of ^68^Ga-Sienna+ in human serum at 37 °C (mean ± SEM, n = 3). (**b**) Cell uptake of ^68^Ga-Sienna+ in breast cancer cells (3E.Δ.NT) and macrophages (J774A.1 and RAW264.7.GFP) at different time points (mean ± SEM, n = 3).

^68^Ga-Sienna+ in human serum at 37 °C showed good radiolabelling stability for at least up to 4 h (84 ± 6 %, Figure 2a). The cell uptake study of ^68^Ga-Sienna+ was performed in the same breast cancer cell line as used in the *in vivo* study (3E.Δ.NT) and with two different macrophage cell lines (J774A.1 and RAW264.7.GFP). In all the cell lines tested, ^68^Ga-Sienna+ uptake increased over time (Figure 2b). Both macrophage cell lines had significantly higher uptake than cancer cells at all time points (*P* < 0.001). Highest uptake (12 ± 1%) was found in J774A.1 cells at 3 h, compared to 2.0 ± 0.1% in cancer cells.

### *In vivo* imaging and histology

**Figure 3:**
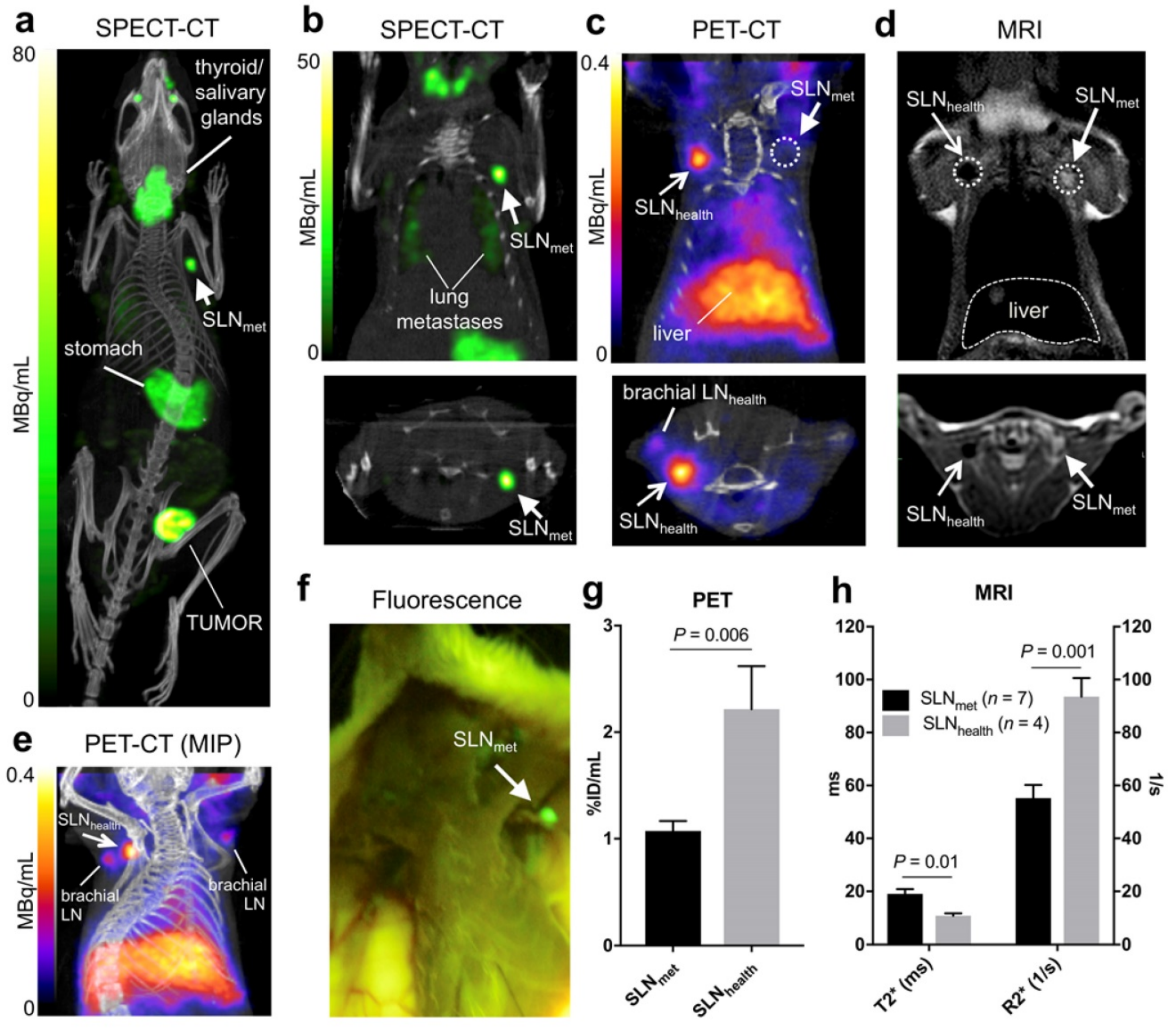
*In vivo* imaging in the 3E.Δ.NT metastatic breast cancer model: (**a**) whole-body MIP SPECT-CT showing uptake of ^99m^TcO_4_^-^ in the primary tumour and left axillary lymph node (SLN_met_, filled arrowhead) due to metastasis, as well as uptake in NIS-expressing organs (thyroid/salivary glands and stomach); (**b**) coronal (top) and transversal (bottom) SPECT-CT images for the same mouse centered in SLN_met_ and showing presence of lung metastases; (**c**) coronal and transversal PET-CT images for the same mouse showing uptake of ^68^Ga-Sienna+ in SLN_health_ (open arrowhead), but not in SLN_met_. Note that, for clarity, the injection site and corresponding PET signal is not shown; (**d**) coronal and transversal MRI images (T2-weighted 3D turbo spin echo) of the same mouse showing the uptake of ^68^Ga-Sienna+ in SLN_health_, but not in SLN_met_ that appear bright and enlarged; (**e**) MIP PET-CT image showing the relative uptake of ^68^Ga-Sienna+ in brachial and axillary SLN_health_. Note that, for clarity, the injection site and corresponding PET signal is not shown; (**f**) Fluorescence photograph of same animal as in A-E showing the presence of metastasis in SLN_met_ but not in the contralateral area where SLN_health_ is located. All images are in right-left orientation; (**g**,**h**) ^68^Ga-Sienna+ uptake quantification (mean ± SEM) in SLN_met_ and SLN_health_ by (**g**) PET and (**h**) MRI.

Six NSG mice were inoculated with 1×10^6^ 3E.Δ.NT breast cancer cells into the left mammary fat pad. After 3 weeks, the primary tumour metastasised into the left axillary lymph nodes (LALNs), as assessed by ^99m^TcO_4_^-^ SPECT (Figures 3a-b). ^99m^TcO_4_^-^ is a substrate for hNIS, which is expressed by the cancer cells 30. Using this method, metastasis in all LALNs could be confirmed. Metastasis was also observed in the lungs (Figure 3b). Organs that endogenously express NIS were also visible (stomach, salivary glands, thyroid and mammary glands) (Figures 3a-b). 3E.Δ.NT cells also express green fluorescent protein (GFP), allowing visual identification and confirmation of LALN metastasis by fluorescence detection during dissection (Figure 3f) and by GFP staining during histological analysis (*vide infra*). In one mouse metastasis was also found in the right axillary lymph node (RALN). In another mouse the health status of the RALN could not be confirmed and was excluded from the analysis. Therefore, in the final analysis, seven metastatic and four healthy LNs were included.

Prior kinetic studies in tumour-bearing mice demonstrated slow SPIO draining (*ca.* 24h) into the axillary SLN when peri/intratumoural injections were used, which would prevent imaging using ^68^Ga. In clinical studies, Sienna+ has shown fast SLN accumulation (<1h); for this reason we evaluated paw injections, a well-proven route of administration of nanoparticulates that results in fast axillary LN accumulation. ^68^Ga-Sienna+ SLN uptake kinetics via paw injection were first assessed in a B6CBAF1 mouse. There was a fast uptake in axillary LN within 20-50 mins that did not considerably change for up to 170 min (data not shown). Therefore, in the 3E.Δ.NT/NSG model, PET scans were performed 1 h after injection of ^68^Ga-Sienna+ into both front paws. MRI was performed 6 h post injection due to the logistics of these experiments. PET imaging showed accumulation of ^68^Ga-Sienna+ in healthy axillary LNs (SLN_health_), as well as - although at a significantly lower uptake level - healthy brachial LNs (Figure 3c and 3e). The high uptake in SLN_health_ allows for differentiation from SLN_met_, which had only background uptake (Figure 3c). Image-based quantification demonstrated a significantly higher uptake in axillary SLN_health_ compared to SLN_met_ (2.2 ± 0.9 *vs.* 1.1 ± 0.2 %ID/mL, *P* = 0.006; Figure 3g). It should be noted that the %ID/mL values for SLN_health_ are likely to be an underestimation of the real value. This is due to their small size that prevented accurate segmentation by CT and the substantial partial volume effect from the PET signal that was used for measuring their volume. ^68^Ga-Sienna+ was also found in the liver (5.3 ± 1.2 %ID/mL), heart (2.1 ± 0.3 %ID/mL), lungs (1.2 ± 0.3 %ID/mL) and bladder (1.5 ± 0.4 %ID/mL) (Figure 3e). In other organs, uptake was below 1 %ID/mL. Most of the injected ^68^Ga-Sienna+ remained in the injection site at the time of the PET scan (67 ± 14 %ID/mL). *Ex vivo* biodistribution studies were not possible due to the timing of the imaging experiments (MRI was performed 6 h after PET imaging), which resulted in decay of the radionuclide.

**Figure 4:**
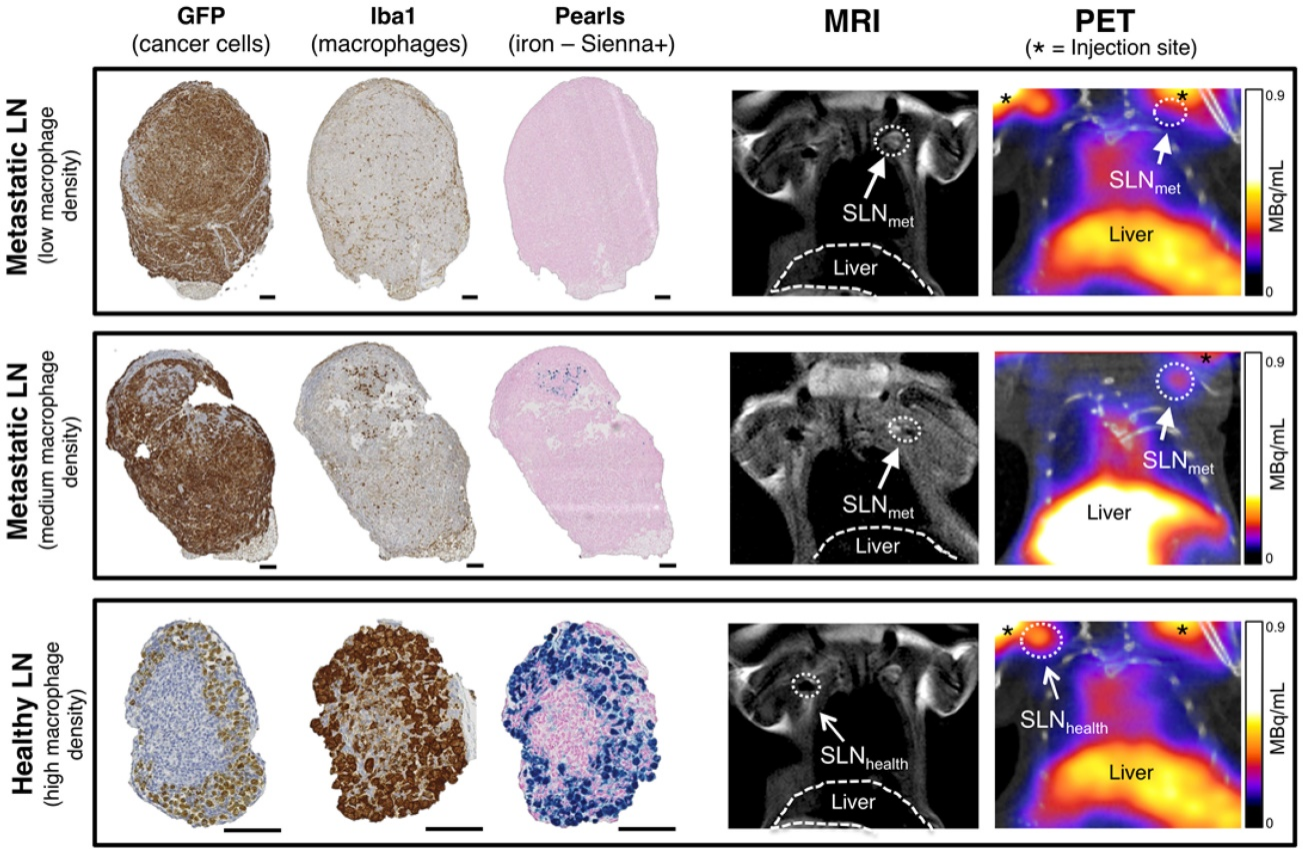
Histological analysis and PET-MRI coronal images of corresponding lymph nodes. Comparison of histology for fully metastatic (top row - with low macrophage density), metastatic (mid-row - medium macrophage density) and healthy lymph nodes (bottom row - high macrophage density) and corresponding MRI and PET-CT images where SLNs are marked with an arrow (SLN_met_: filled arrowhead; SLN_health_: open arrowhead). PET signal from the injection site is marked with an asterisk. Cancer cells (GFP stain) and macrophages (Iba1 stain) stain dark brown, iron (Pearls stain) in blue. Note weak non-specific GFP staining in SLN_health_ to non-cancer cells. Scale bars represent 100 µm.

MRI identified metastasis in SLNs that could not be identified by preclinical PET (Figure 4). SLN_met_ were identifiable in MRI due to their large size and high signal intensity in T2-weighted images due to the absence of ^68^Ga-Sienna+ (Figures 3d and 4). Quantification of the MR images demonstrated these differences with SLN_health_ having significantly lower (T2*)/higher (R2*) values than SLN_met_ (Figure 3h). T2 and R2 were not significantly different between SLN_health_ and SLN_met_, as expected due to the higher sensitivity of T2*-weighted sequences for iron contrast compared to T2-weighted MRI 31.

SLNs from the imaging study with different levels of metastasis and therefore macrophage density were analysed by histology using GFP staining (3E.Δ.NT cells), Iba1 (macrophages) and Perl's Prussian blue (iron/Sienna+) (Figure 4). The location and density of the GFP stain was inversely correlated with that of the macrophage/iron stain and the macrophage-rich areas directly correlated to the iron stain. SLN_met_ were positive for GFP stain whereas healthy SLNs were negative (It should be noted here that there was weak non-specific binding of the GFP stain to SLN_health_, despite absence of GFP-expressing 3E.Δ.NT cancer cells as confirmed by lack of fluorescence). One metastatic SLN_met_ showed a concentrated localization of macrophages and Sienna+ whereas fully metastatic SLN_met_ presented a disperse distribution and low density of macrophages and absence of Sienna+. SLN_health_ showed a high density and co-localization of both macrophages and Sienna+.

## Discussion

The aim of this study was to synthesise and evaluate the potential of ^68^Ga-Sienna+ for locating and characterizing the health status of SLNs with PET-MRI. Sienna+ is a clinically approved SPIO and CE-marked device used in several clinical trials in conjunction with a magnetometer for intrasurgical SLN guidance 32.^ 68^Ga was chosen due to its availability from a GMP-grade generator, its short half-life ideal for SLN imaging, and the possibility of using a chelate-free approach that allows for simple GMP compatible radiolabelling 33-35. Sienna+ nanoparticles were labelled with ^68^Ga without a chelator in a fast and efficient way. A temperature of ≥80 °C was found to be required for efficient labelling. Lower temperatures (40 °C) resulted in RCY below 55%, independently of pH. At high temperatures (100°C) and pH ≥ 6 aggregation occurred. This was prevented by performing the reaction at pH 5. For animal experiments the nanoparticle solution had to be concentrated due to the small volume required for injection (20 µL). This caused a slight increase in the average hydrodynamic particle size, most likely due to minor aggregation as a result of the higher concentration (Table 1). This slight size increase is not expected to result in any significant changes to the pharmacokinetics/biodistribution of Sienna+. Furthermore, when the entire contents of the vial of Sienna+ (2 mL) were radiolabelled (GMP-protocol) the physicochemical properties of the nanoparticle (hydrodynamic size and zeta potential) remained unchanged (Table 1). The highest specific activity achieved in our studies (A_s_ = 67 MBq/mg of Fe) would theoretically allow preparation of up to 4 GBq of ^68^Ga per vial (each vial contains *ca.* 60 mg Fe).

The RCP achieved was within the limits of the European Pharmacopoeia for ^68^Ga radiotracers such as [^68^Ga]Ga-DOTATOC (≥91% RCP) 36, making purification from free ^68^Ga unnecessary. It is not known where exactly ^68^Ga binds to Sienna+, to the coating or the magnetite core. Previous reports using this radiolabelling method, however, suggest radiometal binding to the magnetite core 33. ^68^Ga-Sienna+ was sufficiently stable in serum, with 84 ± 6 % of ^68^Ga-Sienna+ intact after 4h incubation. It should be noted that when used *in vivo,* Sienna+ is injected subcutaneously, and not intravenously. Furthermore, human studies with Sienna+ report fast lymph node uptake, as early as 20 min post injection 37.

As expected from the avidity of dextran-coated SPIOs for macrophages, the *in vitro* cell uptake studies were in agreement with the *in vivo* PET-MRI study, showing a significantly higher uptake of ^68^Ga-Sienna+ in macrophages/SLN_health_ compared to cancer cells/SLN_met_ (Figures 2-3) 38. These findings were confirmed by histology. PET allowed sensitive detection of healthy SLNs (Figure 3e). MRI, on the other hand, allowed the identification of their health status (Figure 3d). Having the complementary information from both PET and MRI and using a bimodal agent allows highly sensitive localization and characterization of SLN and their status at the whole-body level. It is important to mention that, in our preclinical study, partial metastasis could only be detected by MRI by low signal intensity areas (healthy LN tissue) within high signal intensity SLNs (metastatic LN tissue) (Figure 4) whereas by PET these SLNs did not show any measurable signal due to the highly localised [^68^Ga]Ga-Sienna+/macrophage density that is below the spatial resolution limits of preclinical PET due to the partial volume effect. The larger size of human lymph nodes should allow the detection of partial metastasis by both PET and MRI. A likely drawback for the clinical use of ^68^Ga-Sienna+ PET-MRI is that fully metastatic lymph nodes, which do not take up radiolabelled SPIOs, may remain undetected. This could potentially be overcome by the inclusion of diffusion weighted (DW)-MRI into the protocol, which does not depend, but can benefit, from SPIO contrast 39,40.

Looking into how ^68^Ga-Sienna+ could be used for clinical LN imaging, we envisage that after subcutaneous injection of ^68^Ga-Sienna+, in the same way as ^99m^Tc colloids and Sienna+ are currently used for SLNB, patients could undergo a PET-MRI examination where PET will be used first to identify the location of SLN_health_ and partially-metastatic SLN_met_, and second to guide high resolution MRI to these areas for characterization of their health status. This MR step could include DW-MRI in addition to T2/T2*-weighted sequences to identify fully metastatic SLN_met_. One challenge to overcome in order to provide confidence to the use of preoperative SPIO-MRI as a substitute for *ex vivo* histology will be to prevent false positives and negatives. Studies to date using SPIO-MRI in patients have shown a very low number of false-positives (and mostly due to the presence of fatty hilum)^41^. Hence it seems that a more challenging problem will be to prevent false negatives, particularly those involving micrometastases. Indeed, human studies thus far have allowed the detection of LN micrometastases of *ca*. 2mm, and a non-optimal number of false-negatives (*e.g*. 40% of false negatives later identified as micrometastases by histology 41). Several challenges should be overcome to improve this. First, current clinical SPIO/MRI technology does not allow the detection of smaller lesions due to its inherent spatial resolution limitations (potentially sub-millimetre, but restricted by scan time limitations, patient movement and other factors). The second challenge is that the presence of SPIO-related signal in the healthy portion of LNs may obscure small metastatic lesions. To improve this, complementary imaging tests using higher spatial resolution techniques such as photoacoustic imaging 9, and/or improvements in MRI technology (high-resolution 7T magnets, improved coils/sequences for SLN SPIO imaging) or SPIO design (improved relaxation properties and doses), may be able to address these limitations in the future. After imaging, ^68^Ga-Sienna+ could then be detected visually during surgery (or using the standard gamma-probes if ^68^Ga is still detectable) or with the help of a handheld magnetometer, even days after injection 42.

## Conclusion

With the aim of developing a preoperative PET-MRI method to inform sentinel lymph node biopsies (SLNB), we have radiolabelled a clinically-approved SPIO for sentinel lymph node localization (Sienna+) with ^68^Ga using a GMP-compatible, chelate-free method. *In vitro* and *in vivo* studies were performed to confirm the ability of ^68^Ga-Sienna+ in combination with PET-MRI, to locate and characterise SLNs using a breast cancer mouse model that develops spontaneous lymph node metastases. ^68^Ga-Sienna+ uptake levels in SLNs, as measured by PET-MRI followed by histological confirmation, correlate with the level of metastasis/macrophage density. Thus, we have provided proof of concept that ^68^Ga-Sienna+ PET-MRI could be a useful preoperative imaging tool for informing SLNB procedures by allowing the localization at the whole body level (using PET) and characterization (using PET and MRI) of local and distant SLNs from a single imaging session. This would be particularly useful in cancers where the location of the primary tumour, and hence lymphatic drainage, is variable (*e.g.* melanoma). One particular challenge for clinical translation will be the avoidance of false negatives/positives, and it is expected that improvements in MRI/SPIO technology may be able to address this. In future clinical studies, bigger sample sizes and blinded evaluation would be required to confirm the absence of false negatives/positives and to determine cut-off values for image-based quantification and intralymphatic SPIO distribution that would allow accurate prediction of SLN health status in patients.

## Figures and Tables

**Table 1:** Hydrodynamic size (HDS) and zeta potential measurements (mean ± SD) (n = 3)

Sample	HDS (nm) Z-average	HDS (nm) Intensity-weighted	Zeta potential (mV)
**Sienna+**	59 ± 0.7	74 ± 32	-18 ± 3.4
**^68^Ga-Sienna+** (GMP protocol)	57 ± 0.4	71 ± 32	-17 ± 1.2
**^68^Ga-Sienna+** (small scale)	71 ± 0.6	94 ± 48	-11 ± 2.1

## References

[1] Shayan R, Achen MG, Stacker SA (2006). Lymphatic vessels in cancer metastasis: bridging the gaps. Carcinogenesis.

[2] Bader P, Burkhard FC, Markwalder R, Studer UE (2003). Disease progression and survival of patients with positive lymph nodes after radical prostatectomy. Is there a chance of cure?. J Urol.

[3] Balch CM, Soong S-J, Gershenwald JE, Thompson JF, Reintgen DS, Cascinelli N (2001). Prognostic Factors Analysis of 17,600 Melanoma Patients: Melanoma Staging System. J Clin Oncol.

[4] Johnson L, Pinder SE, Douek M (2013). Deposition of superparamagnetic iron-oxide nanoparticles in axillary sentinel lymph nodes following subcutaneous injection. Histopathology.

[5] Kwee TC, Basu S, Torigian DA, Saboury B, Alavi A (2011). Defining the role of modern imaging techniques in assessing lymph nodes for metastasis in cancer: evolving contribution of PET in this setting. Eur J Nucl Med Mol Imaging.

[6] Brouwer OR, Buckle T, Vermeeren L, Klop WMC, Balm AJM, van der Poel HG (2012). Comparing the Hybrid Fluorescent-Radioactive Tracer Indocyanine Green-99mTc-Nanocolloid with 99mTc-Nanocolloid for Sentinel Node Identification: A Validation Study Using Lymphoscintigraphy and SPECT/CT. J Nucl Med.

[7] Heusner TA, Kuemmel S, Hahn S, Koeninger A, Otterbach F, Hamami ME (2009). Diagnostic value of full-dose FDG PET/CT for axillary lymph node staging in breast cancer patients. Eur J Nucl Med Mol Imaging.

[8] Lockau HH, Neuschmelting V, Ogirala A, Vilaseca A, Grimm J (2018). Dynamic 18F-FDG PET- Lymphography for in Vivo Identification of Lymph Node Metastases in Murine Melanoma. J Nucl Med.

[9] Neuschmelting V, Lockau H, Ntziachristos V, Grimm J, Kircher MF (2016). Lymph Node Micrometastases and In-Transit Metastases from Melanoma: In Vivo Detection with Multispectral Optoacoustic Imaging in a Mouse Model. Radiology.

[10] Stoffels I, Morscher S, Helfrich I, Hillen U, Leyh J, Burton NC (2015). Metastatic status of sentinel lymph nodes in melanoma determined noninvasively with multispectral optoacoustic imaging. Immunotherapy.

[11] Harisinghani MG, Barentsz J, Hahn PF, Deserno WM, Tabatabaei S, Hulsbergen van de Kaa C (2003). Noninvasive Detection of Clinically Occult Lymph-Node Metastases in Prostate Cancer. N Engl J Med.

[12] Israël B, van der Linden J, Fortuin AS, Brüggemann R, Scheenen TWJ, Panfilov I (2017). Ultra-small superparamagnetic iron oxides for metastatic lymph node detection: back on the block. Wiley Interdiscip Rev Nanomedicine Nanobiotechnology [Internet].

[13] Pultrum BB, van der Jagt EJ, van Westreenen HL, van Dullemen HM, Kappert P, Groen H (2009). Detection of lymph node metastases with ultrasmall super paramagnetic iron oxide (USPIO)-enhanced magnetic resonance imaging in oesophageal cancer: a feasibility study. Cancer Imaging.

[14] Zbytek B, Carlson JA, Granese J, Ross J, Mihm M, Slominski A (2008). Current concepts of metastasis in melanoma. Expert Rev Dermatol.

[15] Choi J, Park JC, Nah H, Woo S, Oh J, Kim KM (2008). A Hybrid Nanoparticle Probe for Dual-Modality Positron Emission Tomography and Magnetic Resonance Imaging. Angew Chemie Int Ed.

[16] Park JC, Yu MK, An G Il, Park S Il, Oh J, Kim HJ (2010). Facile preparation of a hybrid nanoprobe for triple-modality optical/PET/MR imaging. Small.

[17] T (2011). M. de Rosales R, Tavaré R, Paul RL, Jauregui-Osoro M, Protti A, Glaria A, et al. Synthesis of ^64^Cu^II^- bis(dithiocarbamatebisphosphonate) and its conjugation with superparamagnetic iron oxide nanoparticles: In vivo evaluation as dual-modality PET-MRI agent. Angew Chemie Int Ed.

[18] Madru R, Kjellman P, Olsson F, Wingårdh K, Ingvar C, Ståhlberg F (2012). ^99m^Tc-labeled superparamagnetic iron oxide nanoparticles for multimodality SPECT/MRI of sentinel lymph nodes. J Nucl Med.

[19] Kim JS, Kim Y-H, Kim HJ, Kang KW, Tae EL, Youn H (2012). Development and *in vivo* imaging of a PET/MRI nanoprobe with enhanced NIR fluorescence by dye encapsulation. Nanomedicine.

[20] Madru R, Tran TA, Axelsson J, Ingvar C, Bibic A, Ståhlberg F (2014). ^68^Ga-labeled superparamagnetic iron oxide nanoparticles (SPIONs) for multi-modality PET/MR/Cherenkov luminescence imaging of sentinel lymph nodes. Am J Nucl Med Mol Imaging.

[21] Thorek DLJ, Ulmert D, Diop N-FM, Lupu ME, Doran MG, Huang R (2014). Non-invasive mapping of deep-tissue lymph nodes in live animals using a multimodal PET/MRI nanoparticle. Nat Commun.

[22] Chakravarty R, Valdovinos HF, Chen F, Lewis CM, Ellison PA, Luo H (2014). Intrinsically germanium-69-labeled iron oxide nanoparticles: Synthesis and in-vivo dual-modality PET/MR imaging. Adv Mater.

[23] Jansson T, Andersson-Engels S, Fredriksson S, Ståhlberg F, Strand S-E (2015). Superparamagnetic iron oxide nanoparticles as a multimodal contrast agent for up to five imaging modalities. Clin Trans Imaging.

[24] Kaittanis C, Shaffer TM, Bolaender A, Appelbaum Z, Appelbaum J, Chiosis G (2015). Multifunctional MRI/PET Nanobeacons Derived from the in Situ Self-Assembly of Translational Polymers and Clinical Cargo through Coalescent Intermolecular Forces. Nano Lett.

[25] Yang BY, Moon S-H, Seelam SR, Jeon MJ, Lee Y-S, Lee DS (2015). Development of a multimodal imaging probe by encapsulating iron oxide nanoparticles with functionalized amphiphiles for lymph node imaging. Nanomedicine.

[26] Ko GB, Lee JS, Yoon HS, Kim D, Kim KY, Lee MS (2015). Lymph node imaging using novel simultaneous PET/MRI and dual-modality imaging agent. EJNMMI Phys.

[27] Cui X, Mathe D, Kovács N, Horváth I, Jauregui-Osoro M, Torres Martin De Rosales R (2016). Synthesis, Characterization, and Application of Core-Shell Co0.16Fe2.84O4@NaYF4(Yb, Er) and Fe3O4@NaYF4(Yb, Tm) Nanoparticle as Trimodal (MRI, PET/SPECT, and Optical) Imaging Agents. Bioconjug Chem.

[28] Evertsson M, Kjellman P, Cinthio M, Andersson R, Tran TA, In't Zandt R (2017). Combined Magnetomotive ultrasound, PET/CT, and MR imaging of ^68^Ga-labelled superparamagnetic iron oxide nanoparticles in rat sentinel lymph nodes *in vivo*. Sci Rep.

[29] Edmonds S, Volpe A, Shmeeda H, Parente-Pereira AC, Radia R, Baguña-Torres J (2016). Exploiting the Metal-Chelating Properties of the Drug Cargo for In Vivo Positron Emission Tomography Imaging of Liposomal Nanomedicines. ACS Nano.

[30] Fruhwirth GO, Diocou S, Blower PJ, Ng T, Mullen GED (2014). A Whole-Body Dual-Modality Radionuclide Optical Strategy for Preclinical Imaging of Metastasis and Heterogeneous Treatment Response in Different Microenvironments. J Nucl Med.

[31] Yoshikawa T, Mitchell DG, Hirota S, Ohno Y, Oda K, Maeda T (2006). Gradient- and spin-echo T2-weighted imaging for SPIO-enhanced detection and characterization of focal liver lesions. J Magn Reson Imaging.

[32] Teshome M, Wei C, Hunt KK, Thompson A, Rodriguez K, Mittendorf EA (2016). Use of a Magnetic Tracer for Sentinel Lymph Node Detection in Early-Stage Breast Cancer Patients: A Meta-analysis. Ann Surg Oncol.

[33] Boros E, Bowen AM, Josephson L, Holland JP (2015). Chelate-free metal ion binding and heat-induced radiolabeling of iron oxide nanoparticles. Chem Sci. Royal Society of Chemistry.

[34] Lamb JR, Holland JP (2018). Advanced methods for radiolabeling multimodality nanomedicines for SPECT/MR and PET/MRI. J Nucl Med.

[35] Pellico J, Llop J, Fernández-Barahona I, Bhavesh R, Ruiz-Cabello J, Herranz F (2017). Iron Oxide Nanoradiomaterials: Combining Nanoscale Properties with Radioisotopes for Enhanced Molecular Imaging.

[36] Seemann J, Waldron B, Parker D, Roesch F (2016). DATATOC: a novel conjugate for kit-type 68Ga labelling of TOC at ambient temperature. EJNMMI Radiopharm Chem. EJNMMI Radiopharmacy and Chemistry.

[37] Pouw JJ, Grootendorst MR, Bezooijen R, Klazen CAH, de Bruin WI, Klaase JM (2015). Pre-operative sentinel lymph node localization in breast cancer with superparamagnetic iron oxide MRI: The SentiMAG Multicentre Trial imaging subprotocol. Br J Radiol.

[38] Weissleder R, Nahrendorf M, Pittet MJ (2014). Imaging macrophages with nanoparticles. Nat Mater. Nature Publishing Group.

[39] Ono K, Ochiai R, Yoshida T, Kitagawa M, Omagari J, Kobayashi H (2009). Comparison of diffusion-weighted MRI and 2-[fluorine-18]-fluoro-2-deoxy-D-glucose positron emission tomography (FDG-PET) for detecting primary colorectal cancer and regional lymph node metastases. J Magn Reson Imaging.

[40] Thoeny HC, Triantafyllou M, Birkhaeuser FD, Froehlich JM, Tshering DW, Binser T (2009). Combined Ultrasmall Superparamagnetic Particles of Iron Oxide-Enhanced and Diffusion-Weighted Magnetic Resonance Imaging Reliably Detect Pelvic Lymph Node Metastases in Normal-Sized Nodes of Bladder and Prostate Cancer Patients. Eur Urol.

[41] Motomura K, Ishitobi M, Komoike Y, Koyama H, Noguchi A, Sumino H (2011). SPIO-Enhanced Magnetic Resonance Imaging for the Detection of Metastases in Sentinel Nodes Localized by Computed Tomography Lymphography in Patients with Breast Cancer. Ann Surg Oncol.

[42] Karakatsanis A, Christiansen PM, Fischer L, Hedin C, Pistioli L, Sund M (2016). The Nordic SentiMag trial: a comparison of super paramagnetic iron oxide (SPIO) nanoparticles versus Tc99 and patent blue in the detection of sentinel node (SN) in patients with breast cancer and a meta-analysis of earlier studies. Breast Cancer Res Treat.

