# Supplementary material for: 68Ga-Sienna+ for PET-MRI Guided Sentinel Lymph Node Biopsy: Synthesis and Preclinical Evaluation in a Metastatic Breast Cancer Model
Source: Nanotheranostics. 2019 Jun 6;3(3):255–65. doi: 10.7150/ntno.34727 (PMC6584137; doi:10.7150/ntno.34727)
Supplement: Supplementary file 1 — Supplementary information. [file ntnov03p0255s1.pdf]

Supplemental Information

[^{68}Ga]Ga-Sienna+ for PET-MRI Guided Sentinel Lymph Node Biopsy: Synthesis and Preclinical Evaluation in a Metastatic Breast Cancer Model

Heli Savolainen[1], Alessia Volpe[1], Alkystis Phinikaridou[2], Michael Douek[3], Gilbert Fruhwirth[1] and Rafael T.M. de Rosales[1*]

RAW264.7.GFP macrophages

Raw 264.7 macrophages and 293T cells were purchased from ATCC (via LGC Standards, UK) and cultured as suggested by ATCC. Retrovirus was produced in 239T cells using the pRuf-IRES-GFP plasmid (a kind gift of Andrew Zannettino, University of Adelaide, Australia) together with the packaging plasmids pGP and pVSV-G at mass ratios of 1:1:1, respectively. Transfection of 293T cells was performed using 1 ug/uL polyethylenimine (PEI; linear 25 kDa, Polysciences, UK) at a mass ratio of 3 parts PEI:1part DNA. Virus was harvested 48 h post cell transfection and filtered through 0.45 µm filters (Nalgene, USA) to remove cell debris. Fresh virus was used to infect 10^5 Raw264.7 cells per 6-well (seeded on the evening before transduction) in DMEM containing 10% (v/v) FBS. 48 h post transduction cells were washed three times with DMEM and re-seeded in fresh growth medium. Green fluorescent cells were identified by live cell fluorescence microscopy (EVOS FL, Thermo Fisher Scientific, USA). The mixed macrophage population was expanded and stably transduced Raw267.4.GFP macrophages were isolated by FACS (FACSAria II, BD Biosciences, UK). GFP expression of Raw267.4.GFP macrophages was further assessed by confocal fluorescence microscopy (Leica TCS Sp5, Leica Microsystems, Germany) and immunoblotting. Nuclei were stained with the DNA intercalating dye Hoechst 33342 (Life Technologies, USA) (Supplemental Figure 1A). Rabbit anti-GFP (A-11222, 2 µg/mL, Thermo Fisher Scientific) reveals GFP expression in RAW 264.7.GFP

cells but not in parental cells (Supplemetal Figure 1B). The house keeping protein GAPDH was used as a loading control (mouse anti-GAPDH, GT239, 1 μg/mL, GeneTex, USA).

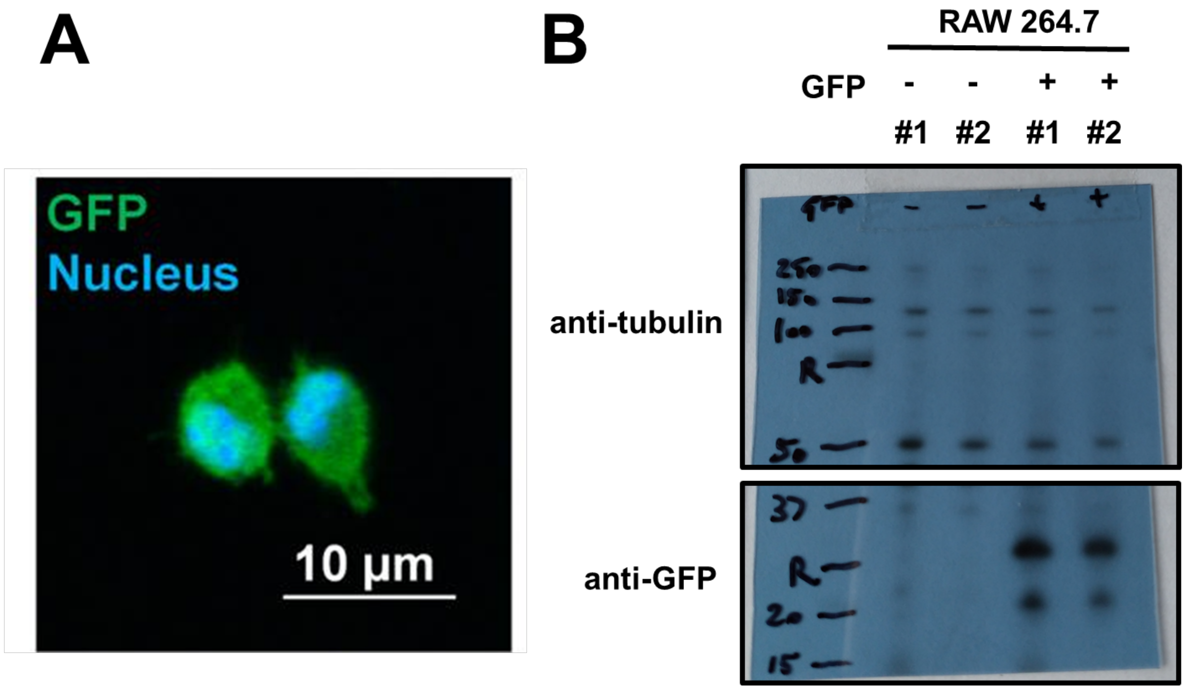

Supplemental Figure 1. (A) Confocal fluorescence microscopy of RAW264.7.GFP macrophages reveals cytosolic GFP distribution (green channel). Nuclei are presented in blue. **(B)** Immunoblot analysis of parental RAW264.7 and stable RAW264.7.GFP cells. Two different cell batches each were lysed and analyzed (#1, #2). The immunoblot was cut in top and bottom halves between the 37 and 50 kDa markers to enable detection of different antigens. The top half was subjected to immuno-detection with anti-tubulin as a loading control (see major band at ~50 kDa with unspecific bands at higher molecular weights). The bottom half was subjected to immuno-detection with anti-GFP demonstrating GFP expression in RAW264.7.GFP cells but not the parental cells (see band at ~27 kDa (“R” = 25 kDa standard marker); the bands at ~ 20 kDa are breakdown product of GFP and usually detected with this antibody, but as expected not present in parental cells). Images shown are photographs without any further processing; the blue-ish color stems from the developed X-ray film. To the left there are marker bands hand-drawn onto the film via blueprinting from the original immunoblot. BioRad’s Precision blue standard was used.
